# A DNA barcode library for the butterflies of North America

**DOI:** 10.7717/peerj.11157

**Published:** 2021-04-19

**Authors:** Jacopo D’Ercole, Vlad Dincă, Paul A. Opler, Norbert Kondla, Christian Schmidt, Jarrett D. Phillips, Robert Robbins, John M. Burns, Scott E. Miller, Nick Grishin, Evgeny V. Zakharov, Jeremy R. DeWaard, Sujeevan Ratnasingham, Paul D.N. Hebert

**Affiliations:** 1 Department of Integrative Biology, University of Guelph, Guelph, Ontario, Canada; 2 Centre for Biodiversity Genomics, University of Guelph, Guelph, Ontario, Canada; 3 Ecology and Genetics Research Unit, University of Oulu, Oulu, Finland; 4 Colorado State University, Fort Collins, CO, United States of America; 5 Unaffiliated, Calgary, Alberta, Canada; 6 Canadian National Collection of Insects, Arachnids and Nematodes, Agriculture and Agri-Food, Guelph, Ontario, Canada; 7 School of Computer Science, University of Guelph, Guelph, Ontario, Canada; 8 Department of Entomology, Smithsonian Institution, Washington DC, United States of America; 9 Department of Biophysics, University of Texas Southwestern Medical Center, Dallas, TX, United States of America; 10 Howard Hughes Medical Institute, University of Texas Southwestern Medical Center, Dallas, United States of America

**Keywords:** DNA barcoding, CO1, Barcode library, Butterflies, North America, Quaternary glaciations

## Abstract

Although the butterflies of North America have received considerable taxonomic attention, overlooked species and instances of hybridization continue to be revealed. The present study assembles a DNA barcode reference library for this fauna to identify groups whose patterns of sequence variation suggest the need for further taxonomic study. Based on 14,626 records from 814 species, DNA barcodes were obtained for 96% of the fauna. The maximum intraspecific distance averaged 1/4 the minimum distance to the nearest neighbor, producing a barcode gap in 76% of the species. Most species (80%) were monophyletic, the others were para- or polyphyletic. Although 15% of currently recognized species shared barcodes, the incidence of such taxa was far higher in regions exposed to Pleistocene glaciations than in those that were ice-free. Nearly 10% of species displayed high intraspecific variation (>2.5%), suggesting the need for further investigation to assess potential cryptic diversity. Aside from aiding the identification of all life stages of North American butterflies, the reference library has provided new perspectives on the incidence of both cryptic and potentially over-split species, setting the stage for future studies that can further explore the evolutionary dynamics of this group.

## Introduction

DNA barcoding is an effective tool for addressing the widely recognized need for an improved understanding of biodiversity. By employing sequence diversity in short, standardized gene regions, such as the 648 base pair segment of the 5′ region of mitochondrial cytochrome *c* oxidase 1 (CO1) employed for the animal kingdom (Hebert et al., 2003), DNA barcoding allows both the identification of specimens and the discovery of new species. Since its introduction, this marker has been adopted in fields ranging from population genetics (Hajibabaei et al., 2007), phylogenetics (Hajibabaei et al., 2007), and phylogeography (Dapporto et al., 2019) to ecology (Valentini, Pompanon & Taberlet, 2009) and conservation (Dincă et al., 2018). It has also gained application in contexts ranging from the detection of marketplace fraud (Galimberti et al., 2013) to the suppression of illegal trade in endangered species (Rehman et al., 2015).

DNA barcoding enables the identification of specimens without morphological analysis by querying their CO1 sequences against a reference library of DNA barcodes obtained from carefully identified vouchers. These reference sequences are curated in the Barcode of Life Datasystem (BOLD) (boldsystems.org) (Ratnasingham & Hebert, 2007), an informatics platform that also hosts collateral data such as specimen images and collection data. Aside from identifying specimens, DNA barcoding can help to delineate species boundaries (Cariou et al., 2020), an important task since species play a central role in biodiversity assessments and conservation actions. Rather than simply presuming that the current taxonomic system is valid, DNA barcoding provides a basis for testing this assertion. Prior studies have shown that closely allied congeneric species of Lepidoptera typically show more than 2% divergence (Hebert et al., 2003; Huemer et al., 2014; Dincă et al., 2015). Although some sister species do show lower divergence (Burns et al., 2007; Cong et al., 2016), cases where species share barcode sequences can reflect over-splitting (Vila et al., 2010) or introgressive hybridization (Zakharov et al., 2009; Cong et al., 2017). Conversely, when members of a putative species show high sequence divergence, this often signals the presence of overlooked species (Hebert et al., 2004; Burns et al., 2007; Dincă et al., 2013). DNA barcode data has also enabled the development of algorithms that employ sequence information for species delimitation. The latter methods cluster specimens into Molecular Operational Taxonomic Units (MOTUs) that have been shown to correspond closely with recognized species in groups with well-established taxonomy (Ratnasingham & Hebert, 2013).

Most studies have tested the capacity of DNA barcodes to discriminate species when viewed from a local or regional context, and only a few have examined resolution at a continental scale (e.g.,  Kerr et al., 2007; Lukhtanov et al., 2009; Bergsten et al., 2012; Huemer et al., 2014; Zahiri et al., 2017; Dincă et al., 2021). The latter studies are important because they can reveal cases of low interspecific divergences, potentially reducing the effectiveness of DNA barcoding for species delimitation. Prior studies have shown the general effectiveness of DNA barcoding for butterflies (Lukhtanov et al., 2009; Dincă et al., 2011; Dincă et al., 2015; Lavinia et al., 2017; Dincă et al., 2021), but have also exposed discordances with current taxonomy including probable cases of synonymy (e.g.,  Vila et al., 2010) and frequent instances of overlooked species (e.g.,  Hebert et al., 2004; Burns et al., 2008).

The butterfly fauna of North America has seen more intensive morphological study than any other comparably diverse insect lineage on this continent (Warren et al., 2012). Despite this attention, there remains uncertainty in the status of many taxa, often reflecting the subjectivity inherent in decisions on species boundaries based on morphology alone. The present study assembles a comprehensive DNA barcode library for the butterfly fauna of North America, delivering an identification system for most of these species while testing the current taxonomy. This work also provides an overview of patterns of genetic diversity and offers insights on mechanisms responsible for shaping the genetic diversity of the butterflies of this continent.

## Methods

### Sampling

This study sought to recover DNA barcodes for the butterfly fauna of Canada and USA. BOLD hosts a checklist for 846 species (CL-NABUT) derived from Pelham’s list (Warren et al., 2012) with a few changes based on recent publications. Table S1 provides a condensed version of this checklist for the 648 species with persistent populations in North America, excluding those introduced by humans.

**Figure 1:**
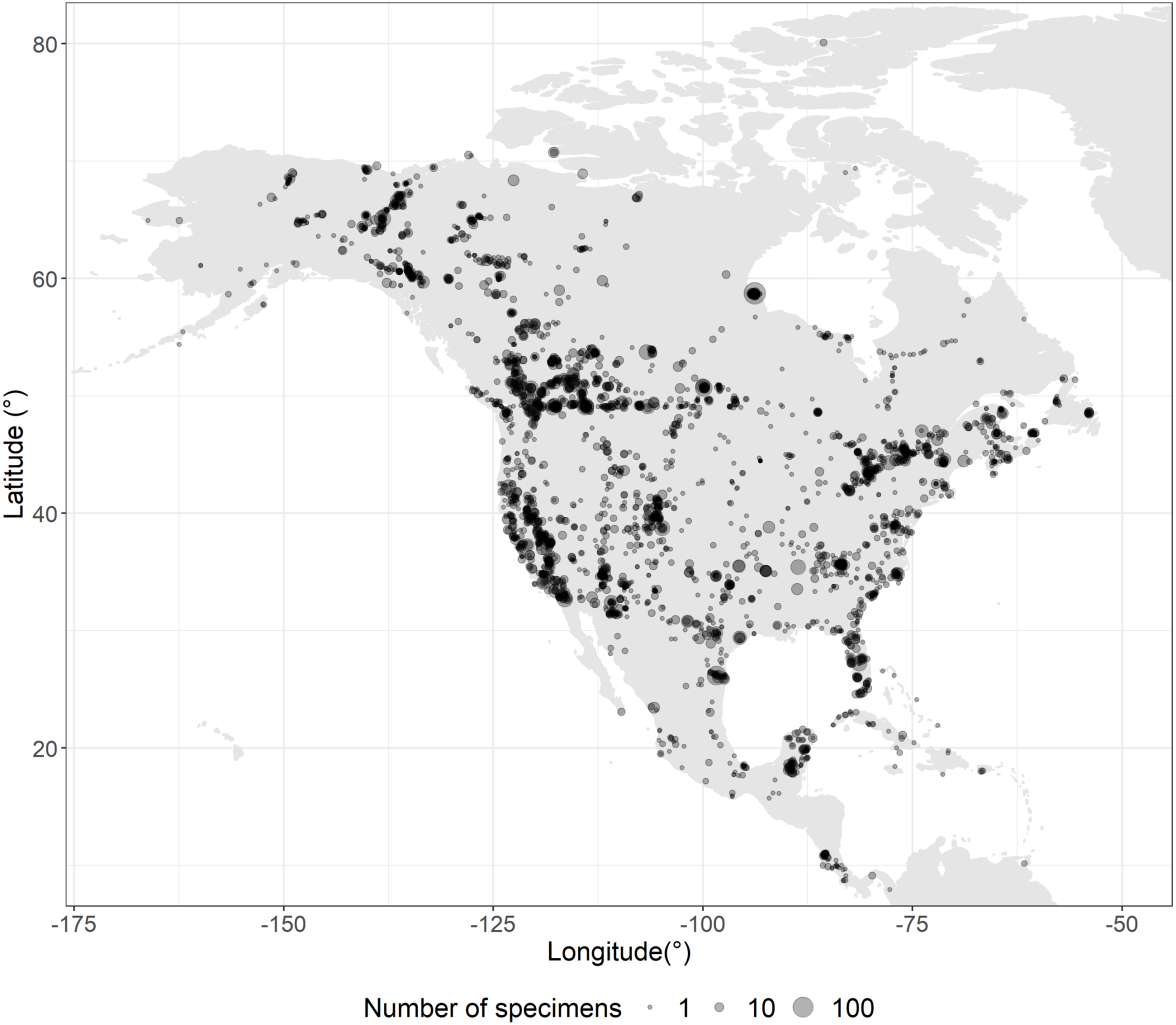
Sampling coverage. Overlapping sampling points are shown as darker circles. Twenty-eight records are not shown in the map as they derived from Argentina, Brazil, China, India, Italy, and Peru.

The sampling program aimed to capture geographic and phylogenetic diversity for each species in continental North America (i.e., islands beyond the continental shelf were excluded). Specimens from Canada (6,935) and USA (7,037) were sequenced when possible, but this left some gaps which were filled by analyzing specimens from Central America (602), South America (27), Europe (7), Asia (4) and unvouchered (11) records from GenBank (Fig. 1). Overall 14,626 specimens were analyzed, and associated metadata are available on BOLD (v4.boldsystems.org) in the public dataset “DS-USCANLEP” (dx.doi.org/10.5883/DS-USCANLEP). From this total, 10,425 vouchers are held in public natural history collections, 3,864 in private collections, 259 derive from GenBank, and 78 were unvouchered. Permission from all the institutional and private collections was obtained to access and study the records. The Centre for Biodiversity Genomics made the largest contribution (4,474 records), followed by the Canadian National Collection (1,771) and the Smithsonian’s National Museum of Natural History (1,010).

iNEXT (INterpolation and EXTrapolation) (Hsieh, Ma & Chao, 2016), was employed to estimate sampling completeness using R (R Studio Team, 2016). This approach implements the Chao1 diversity estimator (Chao, 1984) to generate accumulation curves that can be used to estimate the total haplotype diversity in a species. This asymptotic value was compared with the observed haplotype diversity to quantify sampling completeness, an approach used to estimate coverage for European butterflies (Dincă et al., 2021). Because the present study targeted species resident in North America, levels of genetic diversity for introduced species and tropical strays (for which sampling was limited) were likely to be underestimated. As a result, estimates of sampling completeness excluded species whose distribution primarily falls outside North America. Moreover, species represented by fewer than six specimens were also excluded, reducing consideration to 402 of the 648 species (Table S1). For each species in the barcode library, we recorded the number of specimens (N), the number of observed haplotypes (H), the fraction of haplotype diversity retrieved (R), and the number of additional haplotypes which remain to be sampled (L).

### Mitochondrial CO1 characterization and quality control

DNA extraction, PCR, and sequencing followed standard protocols at the Canadian Centre for DNA Barcoding (CCDB). DNA was extracted using a silica-based method in 96-well plate format (Ivanova, DeWaard & Hebert, 2006). PCR volumes and thermal cycling conditions followed DeWaard et al. (2008) or Hebert et al. (2013) in the case of older museum specimens. Trace files were assembled into contigs with CodonCode Aligner (CodonCode Corporation, http://www.codoncode.com) to generate a sequence record for each specimen. DNA extracts from museum specimens often contain low concentrations of degraded DNA. Although Sanger sequencing can usually recover DNA barcodes from specimens less than 50 years old (Hausmann et al., 2009; Lees et al., 2011; Hebert et al., 2013), sequence length is often <200 bp (Allentoft et al., 2012). Some specimens that failed to generate a Sanger sequence were processed with a next-generation sequencing protocol (D’Ercole, Prosser & Hebert, 2021).

All except five of the 14,626 sequence records included at least 500 unambiguous base pairs of CO1. All sequences were examined for stop codons and the few containing them were removed as likely NUMTS.

Although most specimens were identified by taxonomic specialists through analysis of morphological (i.e., external characters and genitalia) and ecological traits, prior studies have revealed that misidentifications are inevitable in any large-scale study (Mutanen et al., 2016). As a result, Neighbor-Joining (NJ) trees (one for each family) including all records were examined to detect cases where two or more species were admixed in a sequence cluster, a pattern that often arises as a result of misidentification or contamination. The external morphology of specimens in such clusters was examined to confirm their identity, an approach that revealed some errors which were corrected prior to further analyses. Because a detailed inspection (i.e., nuclear markers or genitalic dissections) of all specimens was not possible and because of taxonomic uncertainty in some groups, additional cases of misidentification may remain in our dataset. The NJ tree was also employed to aid the identification of unnamed specimens.

### Genetic analysis

DNA sequences were aligned on BOLD by employing a Hidden Markov Model (Eddy, 1998) based on amino acid sequences. Intraspecific and interspecific genetic distances were calculated using BOLD employing the Kimura two parameter (K2P) distance model (Kimura, 1980). The barcode gap was examined by plotting maximum intraspecific distance for each species against the distance to its nearest neighbor (i.e., minimum interspecific distance). Intraspecific distances and barcode gap analysis could only be calculated for the 755 species represented by two or more specimens.

Bayesian phylogenies (one for each butterfly family) were employed to assess the number of species displaying monophyly. BEAST2 (Bouckaert et al., 2014) was used to generate the trees. The selection of the best model of molecular evolution for phylogenetic investigation was performed with JModeltest2 (Guindon & Gascuel, 2003; Darriba et al., 2012). The GTR model of molecular evolution (Tavaré, 1986), along with the gamma function discretized in four categories, and a parameter for the proportion of invariable sites, were used to estimate genetic distances. Each branch was assumed to evolve at the same rate, accumulating divergence at 1.5% per million years (Quek et al., 2004), following a strict molecular clock. The Markov chain Monte Carlo (MCMC) chain length was 10,000,000 with log frequency every 1,000 samples of the posterior distribution. The pre-burnin was set at 1,000. TreeAnnotator (Bouckaert et al., 2014) was employed to combine the Bayesian trees sampled from the posterior distribution while convergence was confirmed with Tracer (Rambaut et al., 2018). This analysis was restricted to the 755 species represented by two or more specimens as only they could satisfy the definition of monophyly (Hennig, 1966).

The presence of both potentially overlooked and over-split species was tested with three approaches. The first and simplest approach employed a fixed divergence value of 2.5% to discriminate intraspecific from interspecific diversity. Although the application of fixed thresholds is controversial (Collins & Cruickshank, 2013), it provides a useful point of reference for comparison with other studies. The other two methods were the General Mixed Yule Coalescent (GMYC) (Pons et al., 2006; Fujisawa & Barraclough, 2013) and the Barcode Index Number System (BIN) (Ratnasingham & Hebert, 2013). These methods for species delimitation have been shown to perform well in recovering species counts congruent with taxon boundaries established through morphological studies (Ratnasingham & Hebert, 2013). While GMYC typically generates more MOTUs than morphospecies (Miralles & Vences, 2013; Kekkonen & Hebert, 2014), the BIN system was designed to provide a conservative estimate of the number of species (Ratnasingham & Hebert, 2013). The likelihood-based GMYC model makes use of the Bayesian ultrametric trees to determine the transition between intra- and interspecific branching patterns. The R package “splits” (Ezard, Fujisawa & Barraclough, 2009) was employed for the GMYC analysis. A few specimens that were only identified to a generic level were excluded from analysis, and subspecies designations were stripped from specimens that possessed them. The dataset was then collapsed to retain only unique haplotypes (5,116) as past studies showed this approach optimizes results (Talavera, Dincă & Vila, 2013; Tang et al., 2014). BOLD was employed to assign each sequence to a BIN (Ratnasingham & Hebert, 2013). GMYC and BIN assessments generated results falling into four categories: Match, Merge, Split, and Mixture. A species was assigned to the Match category when all of its specimens were assigned to one MOTU. In cases where two or more species shared the same MOTU, they belonged to the Merge category. A species was placed in the Split category when its component specimens were assigned to two or more MOTUs. Finally, a species characterized by a more complex pattern, including both Match and Split, was assigned to the Mixture category.

### Barcode sharing

Barcode sharing describes the situation where individuals of two or more species share identical DNA sequences. As opposed to this, following the character-based definition outlined by DeSalle, Egan & Siddall (2005), species with diagnostic barcodes are those whose sequences show consistent nucleotide differences (or a combination of nucleotide differences) from any other species. As a result, DNA barcodes can be diagnostic even when they derive from species with such low divergence that they are assigned to a single MOTU. To better reflect the differing exposure of species to biogeographic shifts during the Pleistocene, species involved in barcode sharing were partitioned into three categories. The first category (North/alpine hereafter) included species with a geographic distribution north of the last glacial maximum (LGM) and alpine/subalpine species on mountains south of the LGM. The second category (Mid-latitude hereafter) included species with a distribution extending across the LGM. The third category (South hereafter) was composed of species located south of the LGM. The assignment of each species to one of these categories was based on its current distribution (Scott, 1986; Brock & Kaufman, 2006). Because the probability of detecting barcode sharing is influenced by sampling intensity, iNEXT was used to evaluate sampling completeness by region (North/alpine, Mid-latitude, South).

The Spearman’s correlation coefficient was employed to assess the association between the number of species with barcode sharing in a genus and the total number of species in that genus.

## Results

### Sampling and DNA barcoding performance

The present dataset provides barcode coverage for 96.2% (i.e., 814 of 846) of North American butterfly species with an average of 18 sequences per species (Fig. 1, Figs. S1–S6, dx.doi.org/10.5883/DS-USCANLEP). However, 59 species were represented by singletons, including 34 of the 648 species on the truncated list (Table S1). The coverage rises to 97.2% (630 of 648) when only species with permanent populations in North America are considered (Table S1). Estimates of sampling completeness were performed with iNEXT on 63.8% (402 of 630) of the species in the truncated list (Table S1). This analysis, which considered 12,860 specimens, indicated that their 3,212 unique haplotypes corresponded to 67% of the haplotype diversity in this subset of North American butterflies (Table S1). In order to raise haplotype recovery to 100%, it was estimated that at least another 4,702 haplotypes would need to be recovered, an average of 12 haplotypes per species (Table S1).

### Genetic diversity

**Figure 2:**
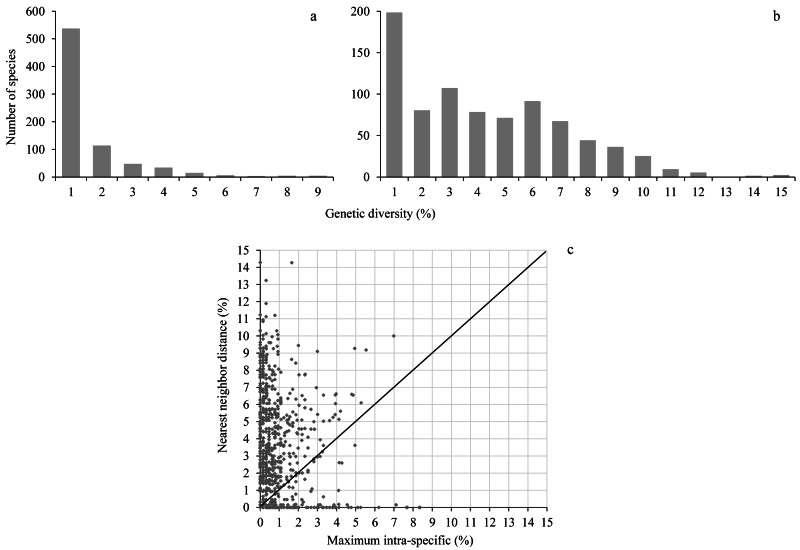
Genetic diversity. The upper histograms show the maximum intraspecific distance (A) and the nearest neighbor distance (B); the lower scatterplot (C) shows for each species (dot) the presence (above the diagonal) or absence (below the diagonal) of a barcode gap.

Maximum intraspecific distances averaged 0.97% (range 0–8.4%) while the nearest neighbor distance averaged 3.7% (range 0–14.3%), almost 4-fold higher than the maximum intraspecific distance (Fig. 2, Table S2). A barcode gap was present in 573 of 755 species (75.9%) represented by at least two individuals (Fig. 2, Table S2).

The Bayesian trees revealed that 604 of the 755 species (80%) represented by two or more individuals formed monophyletic groups, while the other 151 (20%) were either paraphyletic or polyphyletic (Figs. S7–S12, S13 and Table S2). Species with just a single barcode sequence were necessarily excluded from this analysis, but 55 of the 59 possessed barcodes distinct from their nearest neighbor. While discrimination between paraphyly and polyphyly is not essential for specimen identification, it is critical to distinguish those species characterized by overlapping phylogenetic branches from those species sharing barcodes with their nearest neighbor(s). Twenty-six species (3.4%) fell in the first category and 125 (16.6%) species in the latter (Fig. S13).

Use of a fixed distance threshold (2.5%) exposed 79 cases (9.7%) where intraspecific distance was above the set threshold and 324 (39.8%) cases where interspecific divergence was below it. MOTU delineation revealed a variable number of entities depending on the method employed. BIN analysis was performed on all but one species (the sole sequence for *Calephelis rawsoni* did not qualify for analysis) and revealed 772 BINs, comprised of 540 Matches (66.4%), 55 Splits (6.8%), 181 Merges (22.3%), and 37 Mixtures (4.6%). By comparison, GMYC analysis generated 862 taxonomic units, partitioned in 527 Matches (64.7%), 63 Splits (7.8%), 150 Merges (18.4%), and 74 Mixtures (9.1%) (Figs. S7–S12). Overall, the three analyses provided concordant support for 369 species recognized by the current taxonomy, but they also revealed 24 species split into two or more units, 124 grouped with one or more nearest neighbor(s), and 34 in both previous categories. The performance of the three methods is compared in Table S2.

### Barcode sharing

In total, 125 of the 814 (15%) species shared their barcode with another species (Table S2). The incidence of barcode sharing on the condensed list (Table S1) was highest at 42.2% (38/90) for the Northern/alpine species, dropped to 25.6% (55/215) for the Mid-latitude species, and was just 9.2% (30/325) for the Southern species.

iNEXT (Table S1) revealed that the analysis of an average of 41 specimens/species in Northern/alpine species detected an average of 8 unique haplotypes/species, corresponding to 66% of the estimated haplotype diversity for these taxa. By comparison, the analysis of an average of 39 specimens/species for the Mid-latitude species detected an average of 10 haplotypes/species and represented 64% of their estimated diversity. Finally, the analysis of 16 specimens for the Southern species revealed an average of 5 unique haplotypes, corresponding to 71% of their estimated haplotype diversity.

The incidence of barcode sharing varied among butterfly families. The Hesperiidae (21/205, 10.2%) and Riodinidae (2/18, 11.1%) were least impacted, followed by Nymphalidae (30/173, 17.3%), Papilionidae (7/31, 22.6%), Lycaenidae (38/137, 27.7%), and Pieridae (25/66, 37.9%) (Table 1, Table S1). Spearman’s coefficient revealed a positive but non-significant correlation between the incidence of barcode sharing and the number of species in a genus (*R*^2^ = 0.37, *p* = 0.056; Fig. S14).

## Discussion

This study generated a DNA barcode reference library for 96% of the North American butterfly fauna (814 of 846 species) with an average of 18 records per species. This level of sampling captured 67% of the estimated haplotype diversity, but at least 4,705 haplotypes await detection. The present library was effective in assigning newly encountered specimens to either a species or to a small number of closely allied species. Because butterflies are key bioindicators (Syaripuddin, Sing & Wilson, 2015) and umbrella species (New, 1997), this library facilitates their use in tracking the impacts of habitat loss, fragmentation, and climate change. Along with the reference barcodes available for other taxonomic groups (Ratnasingham & Hebert, 2007), the present work also provides a basis for exploring species interactions.

**Table 1:** Distribution of barcode sharing with respect to the LGM by family. Proportions are shown in brackets.

	Above LGM and alpine/subalpine	Across LGM	Below LGM	Total
Papilionidae	2/7 (28.6%)	3/11 (27.3%)	2/13 (15.4%)	7/31 (22.6%)
Pieridae	16/18 (88.9%)	8/15 (53.3%)	1/33 (3%)	25/66 (37.9%)
Hesperiidae	3/11 (27.3%)	10/62 (16.1%)	8/132 (6.1%)	21/205 (10.2%)
Lycaenidae	9/15 (60%)	14/54 (25.9%)	15/68 (22.1%)	38/137 (27.7%)
Riodinidae	0	1/3 (33.3%)	1/15 (6.7%)	2/18 (11.1%)
Nymphalidae	8/39 (20.5%)	19/70 (27.1%)	3/64 (4.7%)	30/173 (17.3%)
Total	38/90 (42.2%)	55/215 (24.9%)	30/325 (9.2%)	123/630 (19.5%)

The sequence records analyzed in this study were mainly obtained during two ways. The first approach involved the collection of fresh material and lasted about two decades (2000–2019), while the latter spanned three years (2015–2017) and consisted of the targeted analysis of specimens held in two major natural history collections—the Smithsonian’s National Museum of Natural History and the Canadian National Collection. Previous studies on Lepidoptera, largely regional in scale, indicated that most species are separated from their nearest neighbor by a barcode gap (e.g., Janzen et al., 2005; Hajibabaei et al., 2006; Lavinia et al., 2017). Increased geographical coverage should lead to higher intraspecific genetic variation as a result of the isolation-by-distance effect (Wright, 1943), and reduced interspecific divergences as more species are analyzed (Avise, 2000). Both factors should reduce the difference between intra- and inter-specific divergence. It needs emphasis that exceptions to this model were observed in butterflies and that narrow suture zones with high diversity alternate with extended regions with little variation (e.g., Gompert et al., 2010; Dapporto et al., 2019; Platania et al., 2020). This continental-scale study revealed that average maximum CO1 divergences within species was nearly four times less than the average minimum distance to the nearest neighbor. Reflecting this fact, 76% of the species displayed a barcode gap ensuring their unambiguous identification. Identification of specimens using the criterion of monophyly raised identification success as 80% of North American butterfly species formed a monophyletic cluster. These results approximate those obtained in a study of European butterflies where monophyly was met for 94% of species at a regional level (Iberia) (*χ*2 = 23.1; *p*-value <0.001) versus 85% for the continent (Dincă et al., 2015) (*χ*2 = 2.71; *p*-value = 0.099). As the European study only considered approximately 60% of the fauna, the proportion of monophyletic species is likely to decline with increased coverage. In a study examining how increased geographic scale affected the barcode gap in Asian butterflies, Lukhtanov et al. (2009) found that the barcode gap decreased with distance, but that species resolution was nearly unaltered. Interestingly, they showed that monophyly, unlike the barcode gap, was less affected by geographic coverage. The evidence for reduced identification success in the present study is likely explained, at least in part, by the 5-fold higher sampling effort (18 specimens/species) than in Lukhtanov et al. (2009) (3 specimens/species). Although the presence of a barcode gap or monophyly are sufficient conditions to ensure the correct assignment of a sequence to its correct species, these are not essential criteria. For example, a species whose component sequences are paraphyletic or polyphyletic can meet neither criteria, but can be perfectly diagnosable (Ross, Murugan & Li, 2008; Bergsten et al., 2012).

Because the capacity of DNA barcoding (Ratnasingham & Hebert, 2007) to deliver a correct identification ultimately depends upon the presence of diagnostic (or a diagnostic combination of) characters, sequence sharing by species indicates that their discrimination will be compromised. This study revealed that 15% of North American butterfly species share their DNA barcodes with another species. Work on European butterflies revealed 3% barcode sharing at a regional level (Iberia), and 7% at a continental scale (Dincă et al., 2015). Although the latter value is about half that observed for North America (*χ*2 = 12.6; *p*-value <0.001), it was inferred based on 60% of the European fauna and more comprehensive taxonomic and geographic coverage will almost certainly increase the incidence of barcode sharing for European butterflies. Because the level of barcode sharing does not only depends on geographic coverage, it is important to ascertain the factors underlying this pattern. First, both the incomplete sorting of ancestral polymorphisms and introgression can lead to barcode sharing, particularly between recently diverged species. The former factor should be less important for mitochondrial than nuclear genes because their lower effective population size facilitates the loss of ancestral polymorphisms. The impact of introgression is more controversial. Although Haldane’s rule predicts that the heterogametic sex (females in Lepidoptera) of hybrid individuals is not likely to pass on mitochondrial DNA (Haldane, 1922), introgression has often been reported in butterflies (e.g., Sperling, 1993; Gompert et al., 2006; Wahlberg et al., 2009). This could be explained by a more general hypothesis, broadly applicable to all organisms, suggesting that low purifying selection on introgressed mitochondrial genes favours the transfer of these elements (over nuclear ones) across species boundaries ( Harrison, 1993). Another feature that facilitates contact between closely related lineages, increasing the likelihood for introgression, is the high dispersal capability of some butterflies (Stevens, Turlure & Baguette, 2010). Second, although butterflies possess a relatively well-established taxonomy, recent studies have exposed taxonomic uncertainty including cases of over-split species (e.g., Vila et al., 2010). Such unrecognized cases of synonymy can produce barcode sharing. Third, although the specimens examined in this study were identified by specialists, DNA barcode results revealed a number of misidentifications. In other cases, the discrimination of morphologically similar species is so difficult that diagnostic characters might have been misinterpreted inflating the incidence of barcode sharing. A full investigation of the roles played by these three factors is beyond the scope of this study, however, an exhaustive study of 42,000 specimens representing nearly 5,000 species of European Lepidoptera showed that just 40% of non-monophyletic species were generated by biological factors (i.e., introgression, incomplete lineage sorting) while 60% reflected methodological problems such as misidentifications (Mutanen et al., 2016).

The incidence of barcode sharing in North American butterflies varies nearly 5-fold with latitude, being far higher among species found in the North/alpine (42%) than at Mid-latitude (25%) or South (9%) locales. A similar pattern was evident for 1541 North American noctuoid moth species as the Canadian fauna showed 10% barcode sharing (Zahiri et al., 2014), versus 7% for the continent (Zahiri et al., 2017).

Although the main scope of this work was to build a comprehensive barcode library for the identification of North American butterflies, effort was made to capture diversity from regions where contact zones, hybrid zones, and phylogeographic breaks congregate (Swenson & Howard, 2005). This strategy aimed to maximize the recovery of haplotype diversity, yet ascertainment bias is inevitable and likely impacted both geography (Yang, Ma & Kreft, 2013) and taxonomy (Troudet et al., 2017). While under-sampling can exaggerate the sequence divergence between species, comprehensive knowledge of intraspecific diversity will decrease interspecific distances and expose barcode sharing (e.g., Wiemers & Fiedler, 2007; Dasmahapatra et al., 2010). Our sample sizes were similar (40 specimens/species) for two regions (North/alpine, Mid-latitude), but lower in the South (16 specimens/species). However, iNEXT indicated that similar proportions of CO1 diversity were recovered from each bioregion (66%—North/alpine, 64%—Mid-latitude, 71%—South). Although iNEXT brings statistical rigor to such estimates, its accuracy closely depends on sampling quality. For instance, under-sampling biodiversity hotspots would give the illusion of low diversity and inflate estimates of sampling completeness. This result suggests that the observed differences in barcode sharing are not an artefact of varied sampling coverage. This pattern could well reflect the different exposure of species in each bioregion to the impacts of Quaternary glaciation (Hewitt, 1996). Species in northern regions could have experienced recurrent cycles of isolation in glacial refugia, leading to subsequent opportunities for secondary contact and mitochondrial exchange (Hewitt, 2000). Moreover, the small size of the populations at the leading edge of the species distribution might aid the fixation of introgressed mitochondria (Kingman, 1982). Another consequence of rapid expansion into deglaciated habitats would be low density at the leading edge. This situation can create difficulties in finding a conspecific mate and can weaken isolating mechanisms (e.g., Shelly & Bailey, 1992; Alatalo et al., 1998; Willis, Ryan & Rosenthal, 2011), leading to heterospecific mating and introgression (e.g., Wirtz, 1999; Randler, 2002). Not only scarcity of conspecifics could favor hybrid formation, but it could also enhance their persistence because of decreased competition with parental populations (Arnold, 1997).

Aside from this latitudinal pattern, barcode sharing varied nearly 4-fold among butterfly families (*χ*2 = 25.13; *p*-value <0.001), from 10% in Hesperiidae to 38% in Pieridae. Interestingly, a similar pattern was also observed at lower taxonomic rank, among genera, where the incidence of barcode sharing showed a weak and non-significant correlation with the number of species in a genus suggesting taxonomic localization. The genus *Colias* (Pieridae) was particularly impacted as all 22 species shared at least one of their barcode sequences with another species (Table S2), perhaps reflecting their recent radiation (Chew & Watt, 2006; Wheat & Watt, 2008). Not only this pattern explains the introgression of haplotypes between hybridizing species such as *C. eurytheme*/*C. phildice* in the eastern USA (Gerould, 1946; Jahner, Shapiro & Forister, 2011), and *C. eurytheme*/*C. eriphyle* in the west (Taylor Jr, 1972), but it also lays the foundation for operational issues such as misidentifications reflecting the unsettled taxonomy of the genus (Wheat & Watt, 2008).

DNA barcoding combined with species delimitation methods enables rapid, cost-effective surveys of biodiversity. While this is particularly beneficial for poorly-studied taxonomic groups, it can also disclose overlooked diversity in well-studied taxa. BIN analysis indicated that ∼11% of North American butterfly species (6.8% Splits, 4.6% Mixture) were split into two or more entities. The incidence of such cases mirrors values (9–12%) reported in prior studies on Lepidoptera (Huemer et al., 2014; Zahiri et al., 2014). GMYC analysis showed an even higher discordance with current taxonomy, showing that about 17% of species (7.8% Splits, 9.1% Mixture) potentially involve overlooked diversity. Interestingly, when the same approach was applied to 60% of European butterfly species, there was even higher discordance (28%) (Dincă et al., 2015). It is probable that the varied habitats in the Mediterranean basin (Blondel et al., 2010), coupled with the presence of southern refugia during the Pleistocene (Schmitt, 2007), created more genetic structure and/or speciation in Europe (Vodă et al., 2016; Dapporto et al., 2019; Scalercio et al., 2020). Employing a fixed divergence threshold (2.5%), about 10% of North American butterfly species exceeded this criterion. Similar values (8–12% with a 2% threshold) have been reported in other studies on Lepidoptera (Huemer et al., 2014; Zahiri et al., 2014). Based on these results, it is likely that a considerable number of cryptic species await description or that Evolutionary Significant Units (ESUs) within species deserve protection (Avise, 1989). Detailed studies (e.g., nuclear genetic, morphological, ecological) (DeSalle, Egan & Siddall, 2005) should be undertaken on these lineages (Polasky & Solow, 1999).

## Conclusion

This study has generated one of the first continental-scale DNA barcode libraries for an entire taxonomic group. Beyond providing an identification system for most (>96%) North American butterflies, it creates the foundation needed to test the current classification. This library also delivers an overview of large-scale patterns of genetic diversity revealing cases of evolutionary interest such as potential hybridization and of importance to biodiversity conservation such as cryptic diversity and evolutionary significant units. As such, this study provides a basis for improving understanding of the mechanisms that have shaped genetic diversity in the North American butterfly fauna.

##  Supplemental Information

10.7717/peerj.11157/supp-1 Figure S1 Neighbor-Joining (NJ) tree for Hesperiidae NJ tree based on sequence variation in the 658 bp barcode region of the cytochrome *c* oxidase 1 gene for 3,588 barcoded specimens.

10.7717/peerj.11157/supp-2 Figure S2 Neighbor-Joining (NJ) tree for Lycaenidae NJ tree based on sequence variation in the 658 bp barcode region of the cytochrome *c* oxidase 1 gene for 3,703 barcoded specimens.

10.7717/peerj.11157/supp-3 Figure S3 Neighbor-Joining (NJ) tree for Nymphalidae NJ tree based on sequence variation in the 658 bp barcode region of the cytochrome *c* oxidase 1 gene for 5,119 barcoded specimens.

10.7717/peerj.11157/supp-4 Figure S4 Neighbor-Joining (NJ) tree for Papilionidae NJ tree based on sequence variation in the 658 bp barcode region of the cytochrome *c* oxidase 1 gene for 484 barcoded specimens.

10.7717/peerj.11157/supp-5 Figure S5 Neighbor-Joining (NJ) tree for Pieridae NJ tree based on sequence variation in the 658 bp barcode region of the cytochrome *c* oxidase 1 gene for 1,520 barcoded specimens.

10.7717/peerj.11157/supp-6 Figure S6 Neighbor-Joining (NJ) tree for Riodinidae NJ tree based on sequence variation in the 658 bp barcode region of the cytochrome *c* oxidase 1 gene for 212 barcoded specimens.

10.7717/peerj.11157/supp-7 Figure S7 Bayesian tree showing GMYC clustering for Hesperiidae Phylogenetic tree based on sequence variation in the 658 bp barcode region of the cytochrome *c* oxidase 1 gene for 1,226 specimens.

10.7717/peerj.11157/supp-8 Figure S8 Bayesian tree showing GMYC clustering for Lycaenidae Phylogenetic tree based on sequence variation in the 658 bp barcode region of the cytochrome *c* oxidase 1 gene for 1,374 specimens.

10.7717/peerj.11157/supp-9 Figure S9 Bayesian tree showing GMYC clustering for Nymphalidae Phylogenetic tree based on sequence variation in the 658 bp barcode region of the cytochrome *c* oxidase 1 gene for 1,583 specimens.

10.7717/peerj.11157/supp-10 Figure S10 Bayesian tree showing GMYC clustering for Papilionidae Phylogenetic tree based on sequence variation in the 658 bp barcode region of the cytochrome *c* oxidase 1 gene for 163 specimens.

10.7717/peerj.11157/supp-11 Figure S11 Bayesian tree showing GMYC clustering for Pieridae Phylogenetic tree based on sequence variation in the 658 bp barcode region of the cytochrome *c* oxidase 1 gene for 659 specimens.

10.7717/peerj.11157/supp-12 Figure S12 Bayesian tree showing GMYC clustering for Riodinidae Phylogenetic tree based on sequence variation in the 658 bp barcode region of the cytochrome *c* oxidase 1 gene for 111 specimens.

10.7717/peerj.11157/supp-13 Figure S13 Taxon clustering The black portion of the “non-monophyly” column represents species with barcode sharing.

10.7717/peerj.11157/supp-14 Figure S14 Spearman’s correlation Correlation between the number of species with barcode sharing in a genus and the total number of species in that genus.

10.7717/peerj.11157/supp-15 Table S1 Reduced checklist Checklist of the 648 butterfly species with resident populations in Canada and/or the USA. Introduced species are excluded. Bold text indicates species sharing barcode sequences with one or several nearest neighbors; N indicates the number of specimens analyzed, H indicates the number of observed unique haplotypes; R indicates the estimated proportion of haplotype diversity; L indicates the haplotypes to be sampled to capture 100% of the total estimated diversity; ND = No data; NC = Not collected. Species were arranged by family and species.

10.7717/peerj.11157/supp-16 Table S2 Species delimitation methods Intraspecific and Nearest-Neighbor (N-N) distances were calculated using the “K80” distance metric; red font indicates intraspecific distance values greater than 2.5%; blue font indicates N-N distances lower than 2.5%; ND = No data; MOTUs were placed in the categories Match, Split, Merge, and Mixture following the definition described in the “Methods” section. Species were arranged by family and species.

## Additional Information and Declarations

### Data Availability

The following information was supplied regarding data availability: Final barcode sequences, including specimen metadata for the 14623 samples are available at the BOLD database: dataset “DS-USCANLEP” (dx.doi.org/10.5883/DS-USCANLEP).
