# Supplementary figures and images for: A DNA barcode library for the butterflies of North America
Source: PeerJ. 2021 Apr 19;9:e11157. doi: 10.7717/peerj.11157 (PMC8061581; doi:10.7717/peerj.11157)

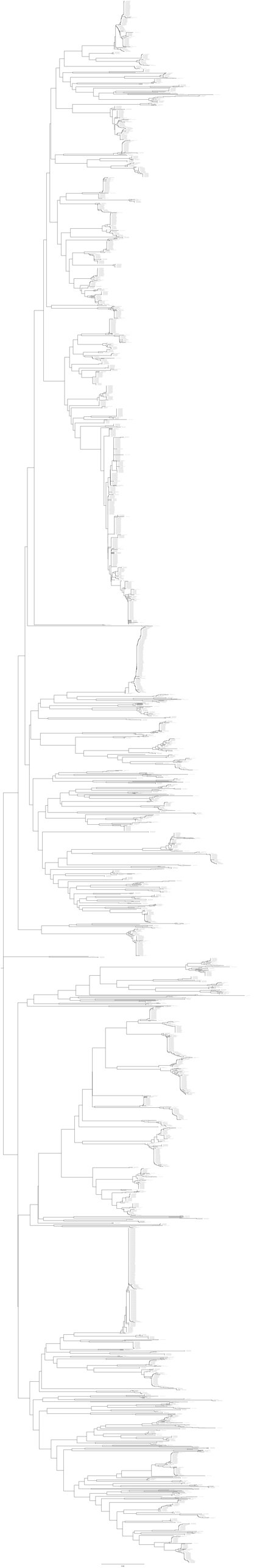

Supplement: Figure S1 — NJ tree based on sequence variation in the 658 bp barcode region of the cytochrome c oxidase 1 gene for 3,588 barcoded specimens. [file peerj-09-11157-s001.pdf]

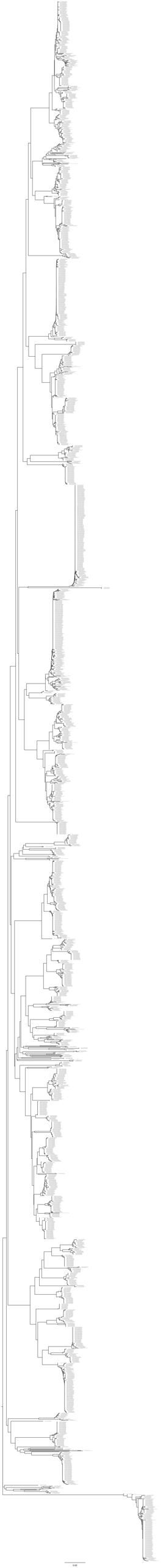

Supplement: Figure S2 — NJ tree based on sequence variation in the 658 bp barcode region of the cytochrome c oxidase 1 gene for 3,703 barcoded specimens. [file peerj-09-11157-s002.pdf]

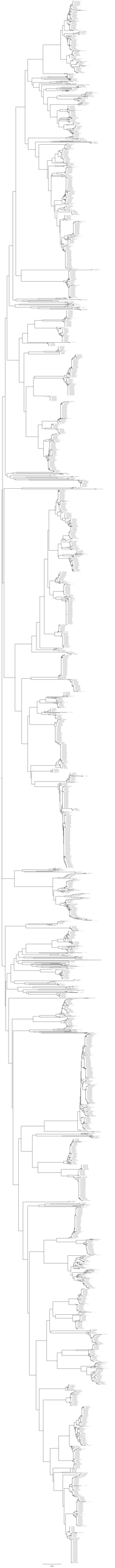

Supplement: Figure S3 — NJ tree based on sequence variation in the 658 bp barcode region of the cytochrome c oxidase 1 gene for 5,119 barcoded specimens. [file peerj-09-11157-s003.pdf]

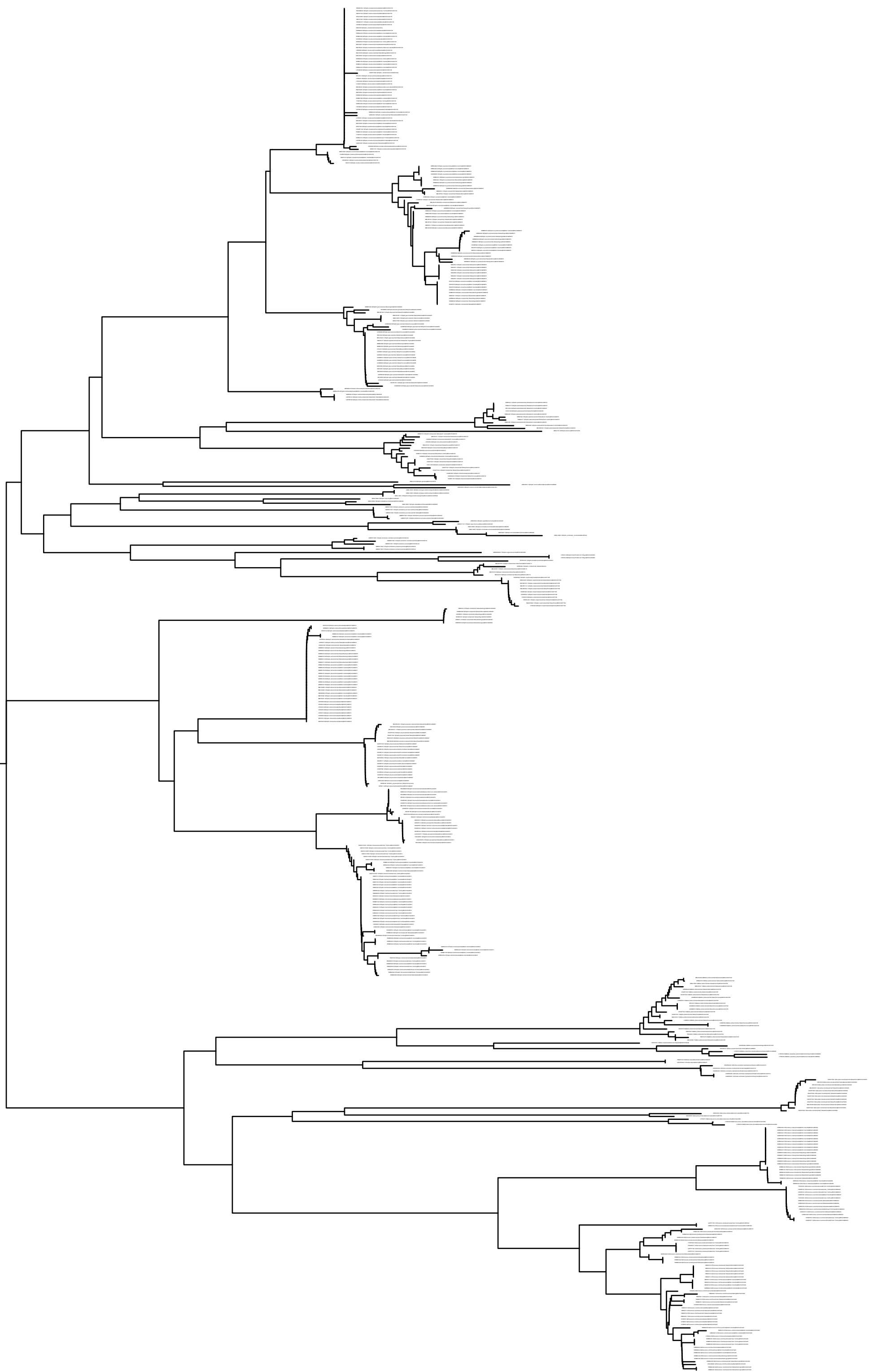

0.01

Supplement: Figure S4 — NJ tree based on sequence variation in the 658 bp barcode region of the cytochrome c oxidase 1 gene for 484 barcoded specimens. [file peerj-09-11157-s004.pdf]

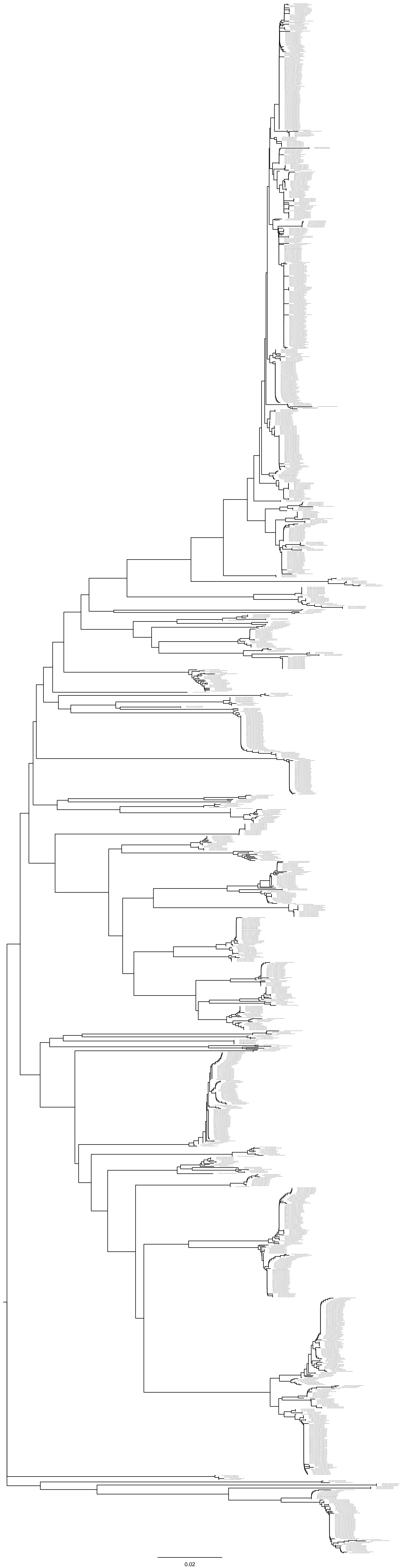

Supplement: Figure S5 — NJ tree based on sequence variation in the 658 bp barcode region of the cytochrome c oxidase 1 gene for 1,520 barcoded specimens. [file peerj-09-11157-s005.pdf]

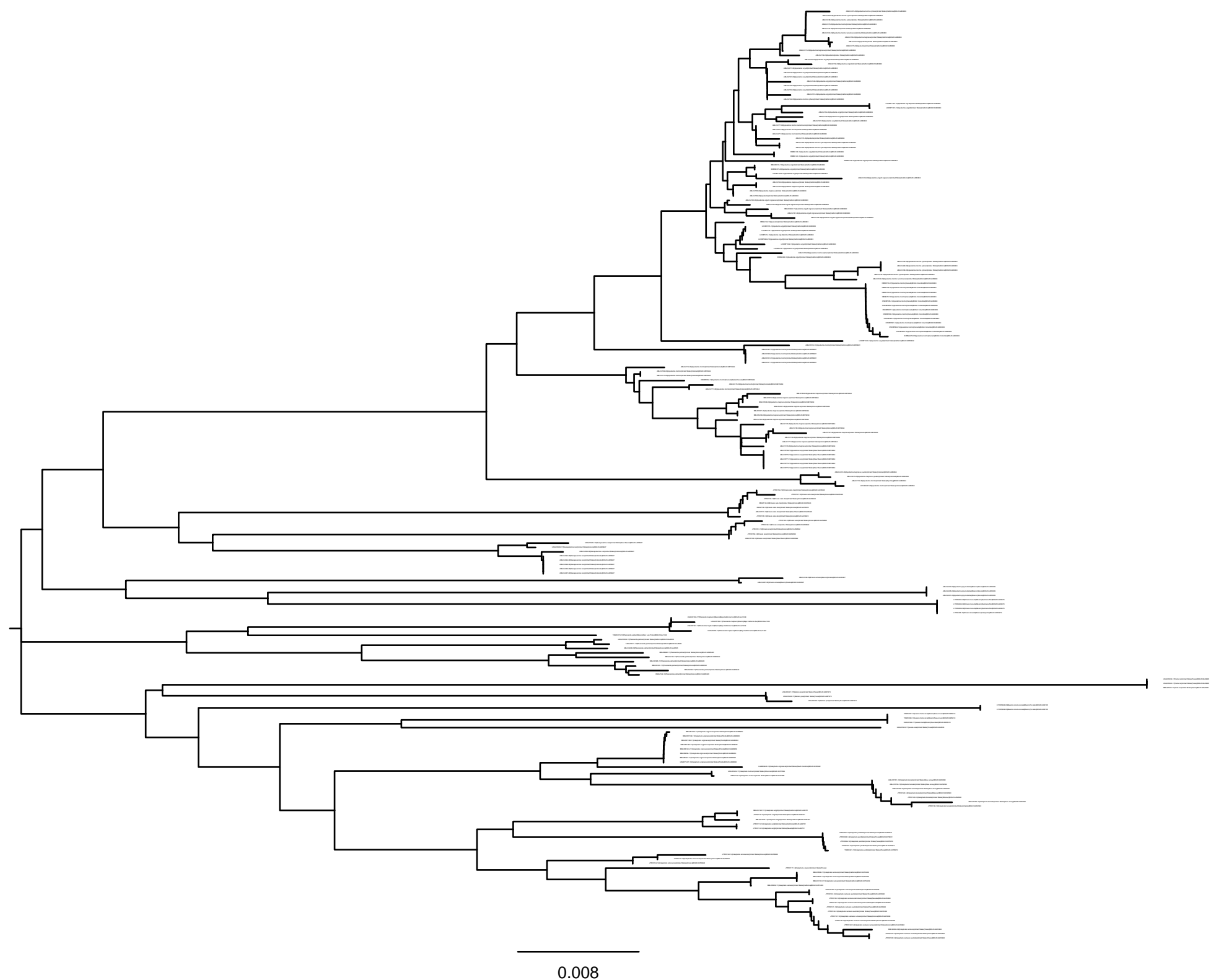

Supplement: Figure S6 — NJ tree based on sequence variation in the 658 bp barcode region of the cytochrome c oxidase 1 gene for 212 barcoded specimens. [file peerj-09-11157-s006.pdf]

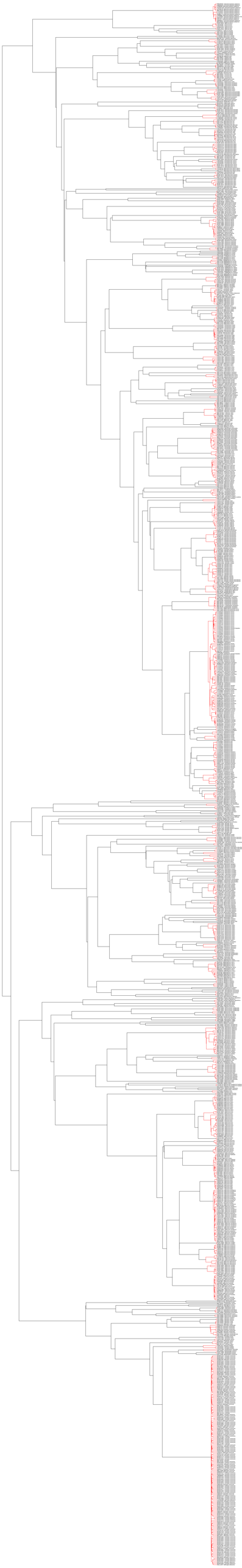

Supplement: Figure S7 — Phylogenetic tree based on sequence variation in the 658 bp barcode region of the cytochrome c oxidase 1 gene for 1,226 specimens. [file peerj-09-11157-s007.pdf]

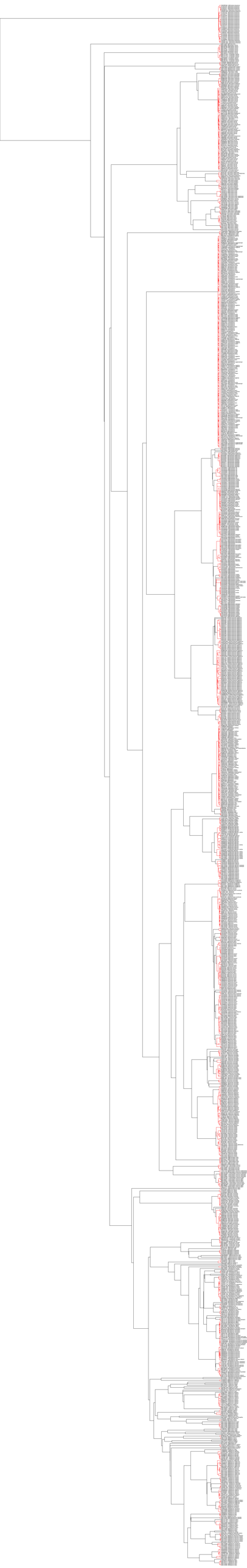

Supplement: Figure S8 — Phylogenetic tree based on sequence variation in the 658 bp barcode region of the cytochrome c oxidase 1 gene for 1,374 specimens. [file peerj-09-11157-s008.pdf]

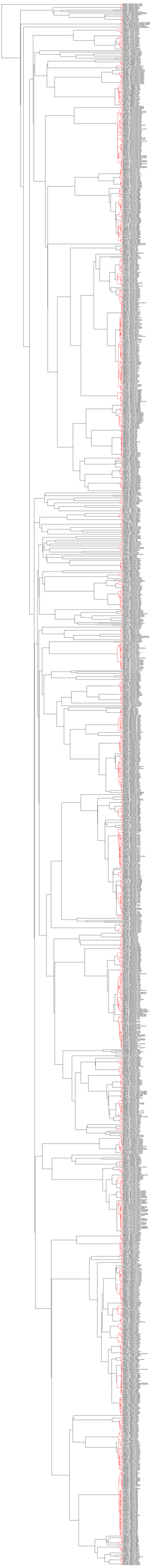

Supplement: Figure S9 — Phylogenetic tree based on sequence variation in the 658 bp barcode region of the cytochrome c oxidase 1 gene for 1,583 specimens. [file peerj-09-11157-s009.pdf]

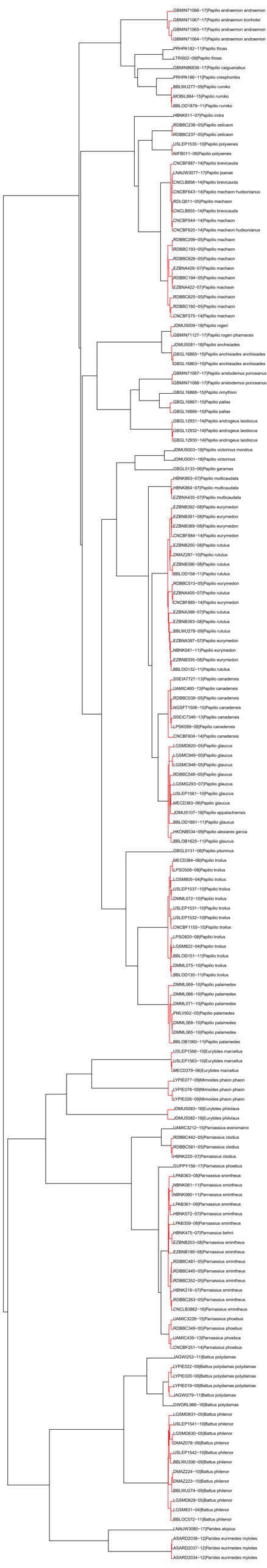

Supplement: Figure S10 — Phylogenetic tree based on sequence variation in the 658 bp barcode region of the cytochrome c oxidase 1 gene for 163 specimens. [file peerj-09-11157-s010.pdf]

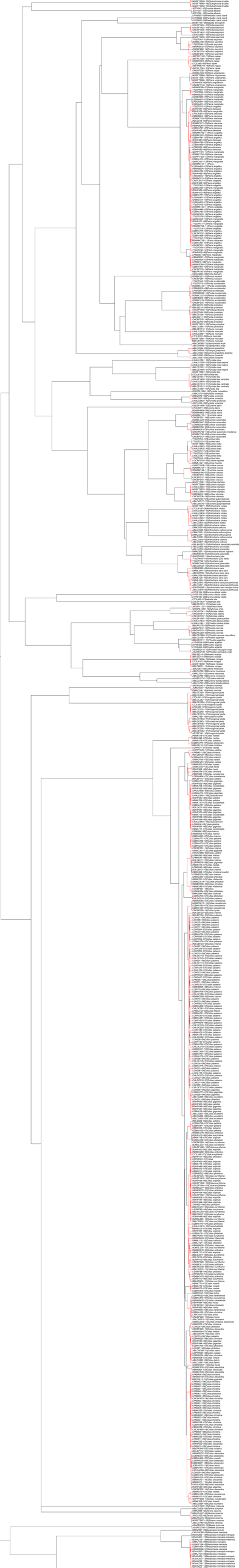

Supplement: Figure S11 — Phylogenetic tree based on sequence variation in the 658 bp barcode region of the cytochrome c oxidase 1 gene for 659 specimens. [file peerj-09-11157-s011.pdf]

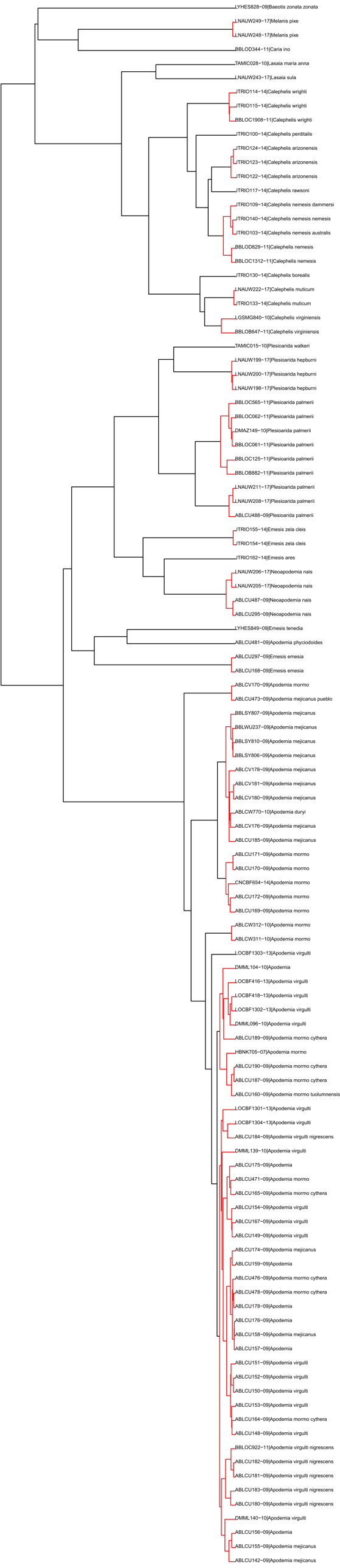

Supplement: Figure S12 — Phylogenetic tree based on sequence variation in the 658 bp barcode region of the cytochrome c oxidase 1 gene for 111 specimens. [file peerj-09-11157-s012.pdf]

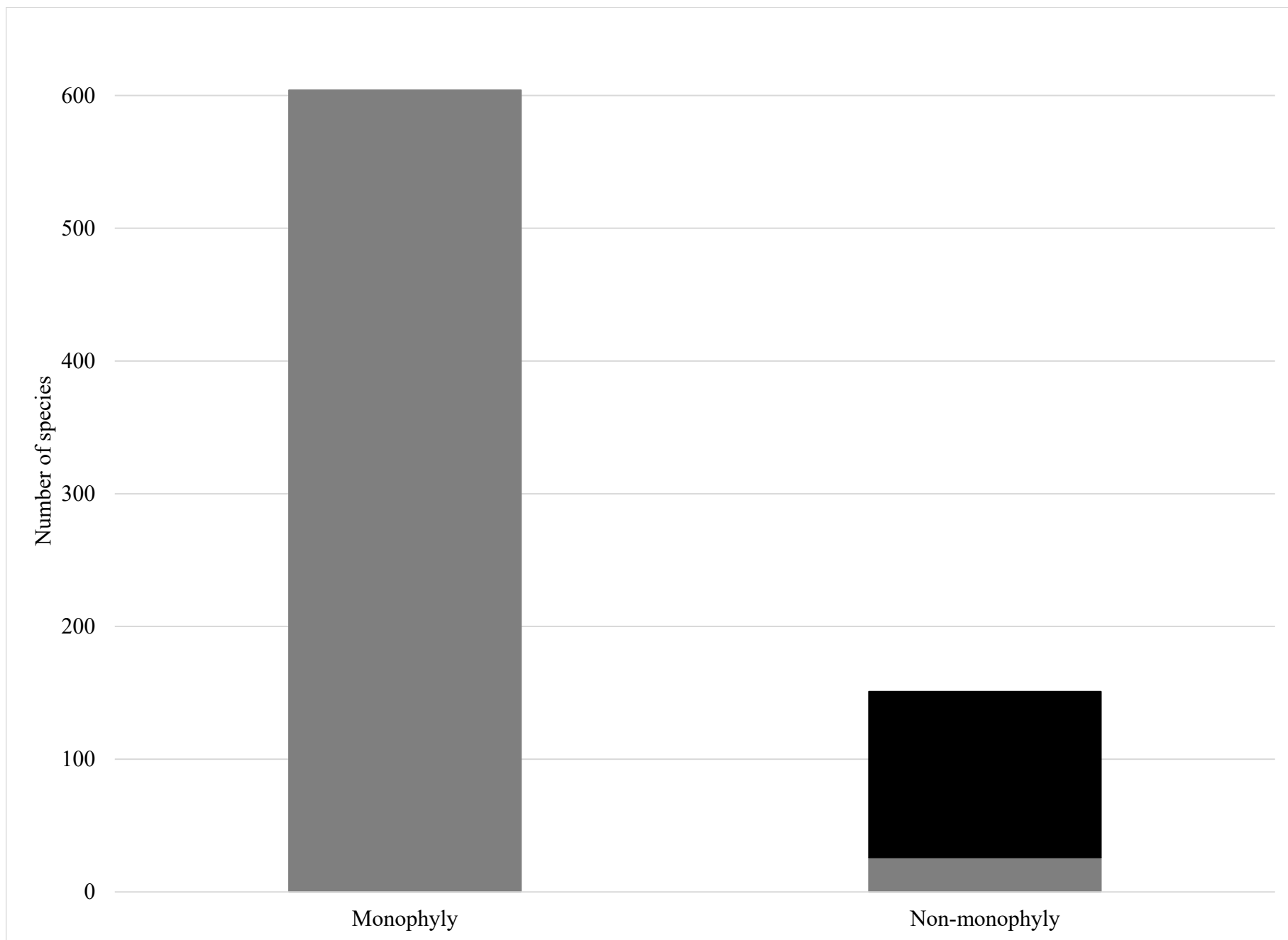

Supplement: Figure S13 — The black portion of the “non-monophyly” column represents species with barcode sharing. [file peerj-09-11157-s013.pdf]

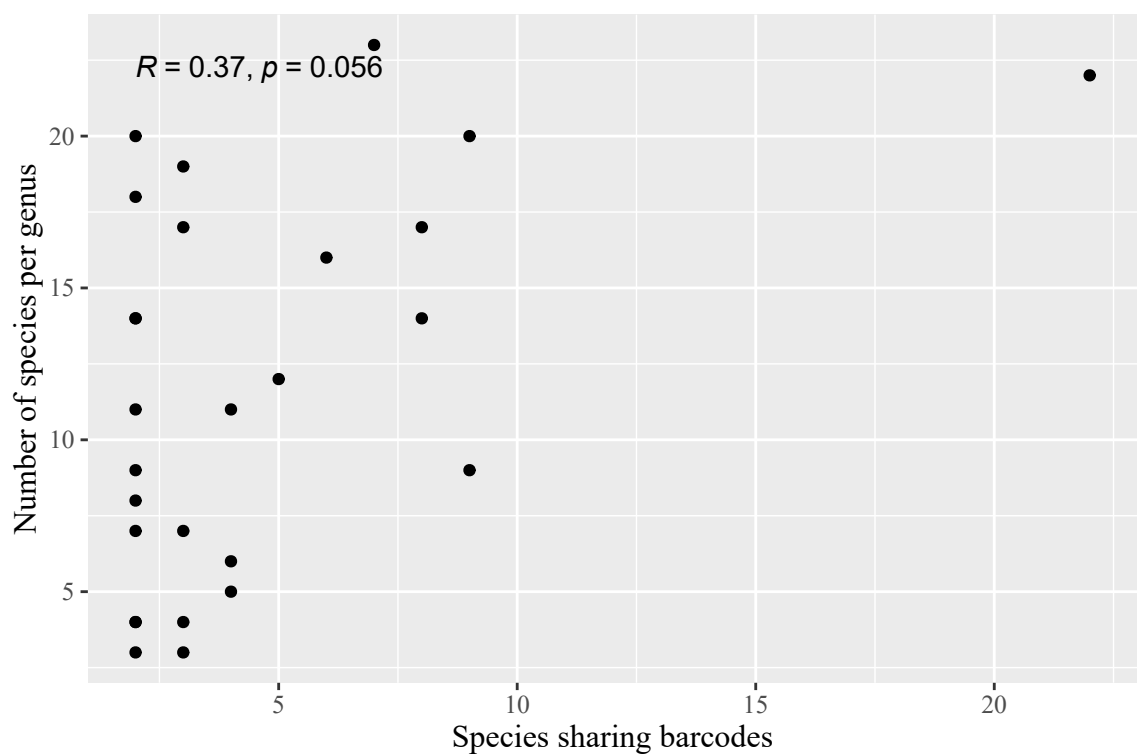

Supplement: Figure S14 — Correlation between the number of species with barcode sharing in a genus and the total number of species in that genus. [file peerj-09-11157-s014.pdf]
